# Associations of Leukocyte Telomere Length With Trait Resilience, Adverse Childhood Experiences, and Psychological Distress Among Expecting Parents in the FinnBrain Birth Cohort

**DOI:** 10.1016/j.bpsgos.2025.100498

**Published:** 2025-04-01

**Authors:** Viivi Mondolin, Hasse Karlsson, Laura Perasto, Tiina Paunio, Emma Vitikainen, Dries S. Martens, Linnea Karlsson, Jetro J. Tuulari, Eeva-Leena Kataja

**Affiliations:** a FinnBrain Birth Cohort Study, Turku Brain and Mind Center, Department of Clinical Medicine, University of Turku, Turku, Finland; b Centre for Population Health Research, Turku University Hospital and University of Turku, Turku, Finland; c Department of Psychology and Speech-Language Pathology, University of Turku, Turku, Finland; d Department of Child Psychiatry, University of Helsinki and Helsinki University Hospital, Helsinki, Finland; e Pediatric Research Center, New Children’s Hospital, University of Helsinki and Helsinki University Hospital, Helsinki, Finland; f Department of Psychiatry, Turku University Hospital and University of Turku, Turku, Finland; g Department of Psychiatry and SleepWell Research Program, University of Helsinki and Helsinki University Hospital, Helsinki, Finland; h Population Research, National Institute for Health and Welfare, Helsinki, Finland; i Centre for Environmental Sciences, Hasselt University, Hasselt, Belgium; j Department of Public Health, Turku University Hospital and University of Turku, Turku, Finland; k Department of Child Psychiatry, Turku University Hospital and University of Turku, Turku, Finland; l Clinical Neurosciences, University of Turku, Turku, Finland; m Turku Brain and Mind Center, Department of Clinical Medicine, Turku University Hospital and University of Turku, Turku, Finland; n Neurocenter, Turku University Hospital, Turku, Finland

**Keywords:** ACE, Anxiety, Depression, Parents, Telomere length, Trait resilience

## Abstract

**Background:**

Telomere attrition has previously been associated with mental health problems and adverse childhood experiences (ACEs). Resilience has been shown to protect against mental health problems even in the context of ACEs. In this study, we examined the associations between leukocyte telomere length (LTL), symptoms of psychological distress, ACEs, and trait resilience. We examined whether LTL mediates the negative effects of ACEs and whether trait resilience moderates the association between LTL and distress.

**Methods:**

The study population was drawn from the ongoing FinnBrain Birth Cohort Study and included 342 mothers and 339 fathers who had provided blood samples and questionnaire data during pregnancy. Questionnaire data included the Connor-Davidson Resilience Scale 10, Edinburgh Postnatal Depression Scale, Symptom Checklist-90, and Trauma and Distress Scale. Data analysis included regression analysis, mixed-methods models, and statistical evaluation.

**Results:**

ACEs were associated with depressive and anxiety symptoms. However, contrary to the initial hypothesis, LTL was not associated with ACEs or distress symptoms and thus did not mediate their association. Furthermore, resilience was not associated with LTL and did not moderate the possible association between LTL and distress symptoms.

**Conclusions:**

We found no association between TL and ACEs, psychological distress, or trait resilience. The mild distress symptoms, limited exposure to high ACEs, and the predominantly moderate to high socioeconomic status in the sample may be relevant to interpreting these findings. Encouragingly, not all ACEs necessarily lead to telomere attrition.

## Plain Language Summary

We examined whether trait resilience, ACEs, or depressive and anxiety symptoms are linked to cellular aging, as measured by leukocyte telomere length, in expectant parents. Trait resilience, a trait-like protective factor, has been associated with stress-related factors, many of which are linked to shorter telomeres; however, the connection between trait resilience and cellular aging remains unknown. Contrary to our hypothesis, we found no significant associations. While previous research has linked shorter telomeres to ACEs and mental health problems, findings have been inconsistent. Our study, conducted in a nonclinical population of parents, provides novel insight into the interrelationships between ACEs, depressive and anxiety symptoms, and trait resilience.

Resilience, defined as the capacity to withstand and recover from adversities, has been shown to positively influence mental health by mitigating stress, depression, and anxiety (1, 2, 3). It has also emerged as a protective factor against the detrimental effects of adverse childhood experiences (ACEs) (4, 5, 6). Resilience appears to be a multifaceted phenomenon, with various approaches to its conceptualization and measurement in research. One approach, referred to as trait resilience, is the focus of the current study. It is considered to be a stable, trait-like characteristic that serves as a protective mechanism that enables individuals to endure and manage hardships (2,7,8). In recent years, interest in the biological factors linked to resilience has grown. Several studies have reported moderate heritability, although definitions of resilience vary widely (9,10). A comprehensive review outlined the biological findings relevant to resilience, highlighting its complexity within a biopsychosocial model (11). Many biological factors in stress response systems, including the functioning of the hypothalamic-pituitary-adrenal axis, amygdala, prefrontal cortex, and sympathetic nervous system activity, have been linked to resilience (11).

One biological marker that has been found to be associated with resilience is cellular aging, particularly telomere length (TL) (12,13). Telomeres are repetitive DNA sequences located at the ends of chromosomes, which serve as protective caps that prevent chromosomal deterioration (14). TL declines with age and is predictive of mortality (15), making it a widely used indicator of biological aging. Furthermore, shorter TL has been associated with psychiatric disorders and psychosocial stress (16,17). In addition, studies have suggested an association between ACEs and shorter TL (18, 19, 20). ACEs encompass a broad spectrum of adverse experiences, including abuse, neglect, and other potentially traumatic events that occur before age 18 years (21). ACEs have been consistently associated with various health outcomes, including somatic diseases, psychiatric disorders, and psychosocial stress (22, 23, 24). Because ACEs may accelerate cellular aging, shortened TL may mediate the effect of ACEs on mental health (25,26). However, findings in this area have been heterogeneous, with some studies showing no association between TL and ACEs (18,27,28).

The association between resilience and TL merits additional investigation, given the links between TL, ACEs, and psychiatric symptoms, all of which have been correlated with lower resilience. Furthermore, trait resilience has been observed to demonstrate stability over time (29). The few studies that have examined the relationship between resilience and TL have used varying definitions of resilience. For example, a study found that resilience, operationalized as a combination of optimism, trait positive affect, active coping, and low perceived stress, was associated with longer telomeres in adults ages 45 to 85 years (12). Another study identified multisystem resilience as a moderating factor in the relationship between major depressive disorder and TL among adults ages 45 to 90 years (13).

In the current study, we focus on a sample comprising young to middle-aged expecting parents, with a mean age of 30.4 for mothers and 32.4 for fathers. Psychological distress, depression, and anxiety disorders are common among mothers and fathers during this period (30, 31, 32, 33, 34). A limited number of perinatal studies have examined the relationship between telomeres, distress symptoms, and ACEs. One recent study identified a correlation between peripartum depression (PPD) and shorter telomeres in pregnant women (35). Furthermore, this study noted that as the number of ACEs increased, individuals who presented with PPD symptoms exhibited shorter telomeres, whereas the opposite was observed in the control group (35). Studies have shown a positive association between resilience and improved mental health during the perinatal period (36, 37, 38, 39, 40). However, to the best of our knowledge, no previous studies have examined the relationship between resilience and TL in parents during the perinatal period.

In this study, our objective was to investigate the relationships between leukocyte TL (LTL), ACEs, symptoms of psychological distress (depressive and anxiety symptoms), and trait resilience. We hypothesized that higher levels of depressive and anxiety symptoms would be associated with shorter LTL, that ACEs would be associated with shorter LTL, and that LTL would mediate the negative effect of ACEs on distress symptoms. We also assumed that higher trait resilience would be associated with longer LTL and that trait resilience would moderate the association between LTL and distress symptoms.

## Methods and Materials

This study is part of the ongoing FinnBrain Birth Cohort Study (http://www.finnbrain.fi), which is aimed at examining the impact of early-life stress, such as prenatal distress, on children’s brain development and overall health over time. The study protocol was approved by the Ethics Committee of the Hospital District of Southwest Finland.

### Participants

**Figure 1:**
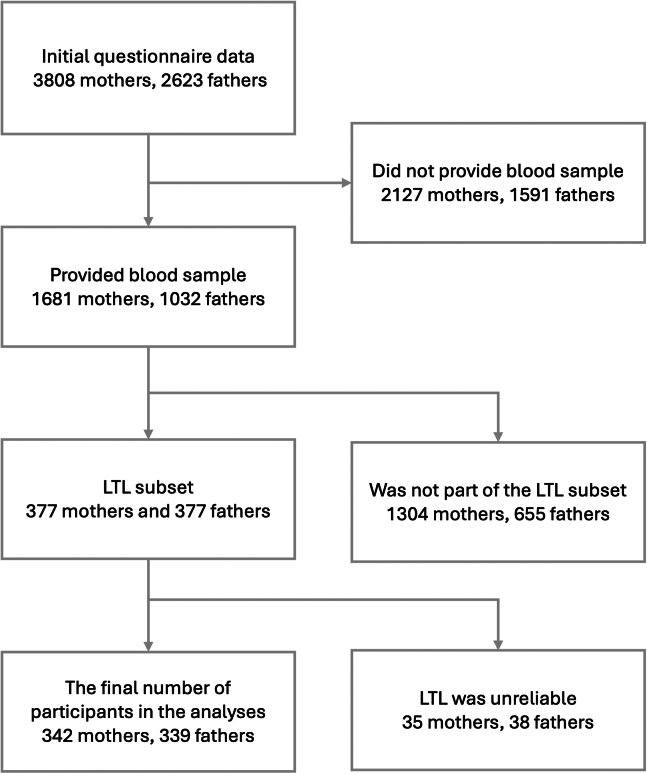
The flowchart illustrates the participant selection process for the current study. LTL, leukocyte telomere length.

The participants of the initial cohort were recruited at 3 maternal welfare clinics in southwestern Finland during their first ultrasound appointment at gestation week (gwk) 12 between December 2011 and April 2015. Inclusion criteria required a sufficient knowledge of Finnish or Swedish (the official languages of Finland) and a normal ultrasound result. Initially, questionnaire data were collected from 3808 mothers and 2623 fathers during pregnancy. Providing a blood sample was voluntary, and samples were collected during a separate laboratory visit at gwk 24. The study cohort was predominantly White and ethnically homogeneous and broadly representative of the expectant parent population in Southwest Finland (41). To be included in the current study, individuals were required to have provided a blood sample for LTL analysis and to have completed symptom questionnaires during pregnancy. Blood samples were collected from 1681 mothers and 1032 fathers. Leukocyte telomeres were isolated from blood samples of 377 mothers and 377 fathers, who represented a subset of the total blood samples. Among these, the samples of 35 mothers and 38 fathers were considered unreliable and were therefore excluded. As a result, 342 mothers and 339 fathers were finally included in this study. The participant selection process is illustrated in Figure 1. The characteristics of the study sample are presented in Table 1. Data from pregnancy questionnaires were used, with details provided in the Measures section. Background variables, including age, educational level, and monthly income, were derived from the questionnaires completed at gwk 14. Education levels were categorized as <12 years, 12 to 15 years, and ≥15 years of education. Monthly net income (in €) was grouped into 4 categories: <1500, 1501 to 2500, 2501 to 3500, and >3500. Missing data were minimal, with the highest proportion being 4% (see Table 1 for details). Given the low percentage of missing values, we decided not to apply imputation.

**Table 1:** Characteristics of the Study Sample

	Mothers, *n*^a^	Mothers			Fathers, *n*^a^	Fathers		
Age, Years	348	30.4 (4.4) [19–42]			327	32.4 (5.9) [20–60]		
Education, Years	337				327			
<12 years	–	32.6%			–	44.6%		
12–15 years	–	31.8%			–	29.4%		
>15 years	–	35.6%			–	26.0%		
Income, €/Month	336				327			
≤1500	–	36.3%			–	20.2%		
1501–2500	–	55.1%			–	56.6%		
2501–3500	–	7.4%			–	17.4%		
>3500	–	1.2%			–	5.8%		
Questionnaire Scores								
Trait resilience	335	28.1 (4.6) [15–39]			326	29.3 (5.1) [12–40]		
Depressive symptoms	346	4.9 (3.6) [0–19]			339	3.2 (2.8) [0–16]		
Anxiety symptoms	346	3.7 (3.6) [0–20]			340	2.2 (2.8) [0–15]		
ACEs	334	10.8 (11.9) [0–86]			325	9.9 (9.2) [0–51]		
Leukocyte telomere length	342	0.85 (0.2) [0.45–1.77]			339	0.87 (0.2) [0.47–1.72]		
ACE Subscales, *n*		Mothers, No. of ACE Scores				Fathers, No. of ACE Scores		

	0	1	≥2		0	1	≥2

Emotional Neglect	–	69	58	202	–	48	46	225
Emotional Abuse	–	134	47	148	–	151	61	107
Physical Neglect	–	95	67	167	–	59	67	193
Physical Abuse	–	180	45	10	–	155	58	106
Sexual Abuse	–	280	17	32	–	304	8	7

Values are presented as mean (SD) [range], *n*, or %. Trait resilience was measured using the Connor-Davidson Resilience Scale 10, depressive symptoms using the Edinburgh Postnatal Depression Scale, anxiety symptoms using the Symptom Checklist-90 anxiety subscale, and ACEs using the Trauma and Distress Scale. Missing data were minimal, ranging from 0% to 4%.

ACE, adverse childhood experience.

a Due to the exclusion of participants with unreliable telomere measurements, sample sizes (*n*) are larger for some variables than in the final telomere analyses.

An attrition analysis was conducted to compare parents included in the current study with individuals in the original cohort, examining age, education, income, trait resilience, depressive and anxiety symptoms, and ACEs for both mothers and fathers. Only fathers’ depressive and anxiety symptoms showed significant differences. Fathers in the original cohort reported higher depressive symptoms (mean = 3.7, SD = 3.2) than fathers in the current study (mean = 3.2, SD = 2.8; *t*_529_ = 2.78, *p* < .01). Similarly, fathers’ anxiety symptoms were higher in the original cohort (mean = 2.53, SD = 3.46) than in the current study (mean = 2.2, SD = 2.8; *t*_563_ = 2.10, *p* < .05). However, these differences were not clinically significant.

### Measures

**Figure 2:**
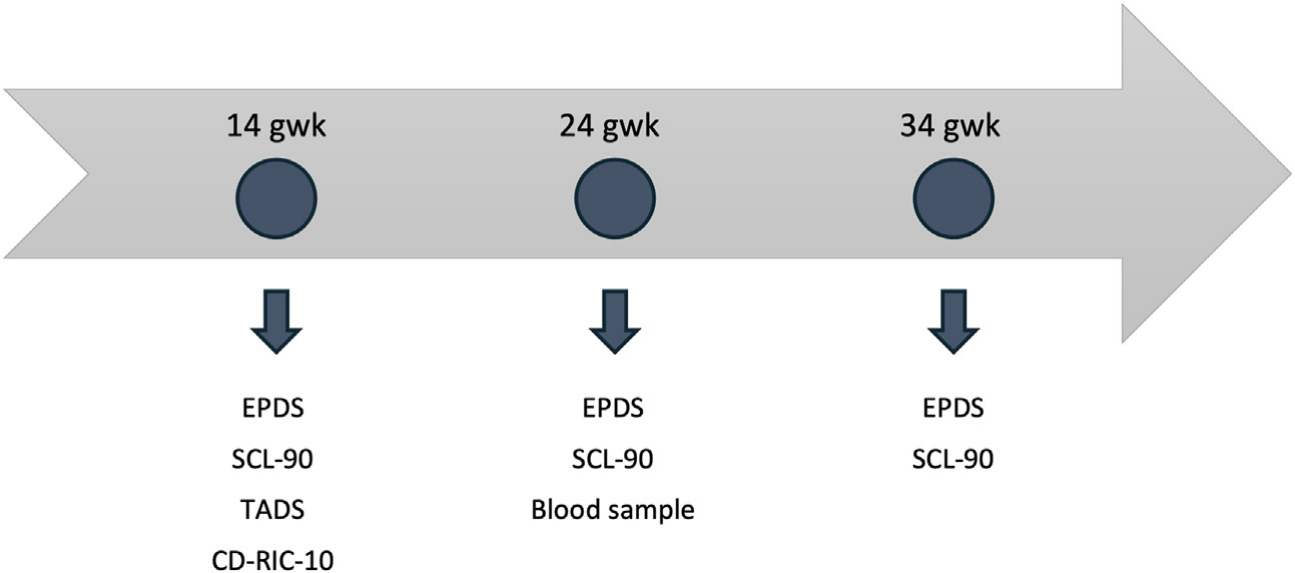
The arrow presents the data collection points of the investigated main variables, which included depressive symptoms (EPDS), anxiety symptoms (SCL-90), adverse childhood experiences, TADS, trait resilience (CD-RISC-10), and leukocyte telomere length (blood sample). The EPDS and SCL-90 were used in analysis as a mean of the score from the 3 different time points. CD-RISC-10, Connor-Davidson Resilience Scale 10; EPDS, Edinburgh Postnatal Depression Scale; gwk, gestation week; SCL-90, Symptom Checklist-90; TADS, Trauma and Distress Scale.

The data collection points, including different questionnaires and the blood sample, are presented in Figure 2.

#### Questionnaire Data

Trait resilience was evaluated using the Connor-Davidson Resilience Scale 10 (CD-RISC-10), a well-validated self-report instrument that has demonstrated strong psychometric properties (7,42,43). The scale consists of 10 items that assess a person’s ability to respond to difficulties and persist in the face of setbacks, rated on a 5-point Likert scale (0–4). The total score, which ranges from 0 to 40, is the sum of the individual item scores, with higher scores indicating greater resilience. The CD-RISC-10 was administered at gwk 14. It showed robust internal consistency (α = 0.83 for mothers, α = 0.86 for fathers).

Parent’s experiences of ACEs were evaluated using the Trauma and Distress Scale (TADS). The TADS was developed to evaluate a range of childhood trauma and distressing experiences, including emotional or physical abuse, sexual abuse, and emotional or physical neglect, through retrospective accounts (44). The TADS comprises 43 items, with the frequency of exposure to maltreatment rated on a 5-point Likert scale ranging from “never” (0) to “almost always” (4). The TADS was administered at gwk 14. The TADS showed good internal consistency reliability, with α values of 0.91 for mothers and 0.89 for fathers.

The psychological distress experienced by both mothers and fathers was evaluated using questionnaires to assess symptoms of depression and anxiety during pregnancy, specifically at gwk 14, 24, and 34. The Edinburgh Postnatal Depression Scale (EPDS) (45), a validated tool designed to detect antenatal depression across various cultural and geographical settings (46,47), was used to assess depressive symptoms. The questionnaire comprises 10 items scored on a 4-point Likert scale (0–3), with total scores ranging from 0 to 30. The internal consistency of the EPDS was satisfactory, with α values ranging between 0.81 and 0.85 for mothers and 0.79 and 0.78 for fathers. The Symptom Checklist-90 (SCL-90) was used to measure anxiety symptoms, with a particular focus on the anxiety subscale, which comprises 10 items (48). Participants rated items on a 5-point Likert scale (0–4), with total scores ranging from 0 to 40 points. The reliability of this subscale has been validated in the Finnish population (49). The SCL-90 demonstrated satisfactory internal consistency, with α values of 0.83 to 0.85 for mothers and 0.80 to 0.83 for fathers. The scores for both depressive and anxiety symptoms were calculated by creating an average variable from the 3 different scores collected during pregnancy.

#### DNA Extraction and LTL

DNA was extracted using Chemagen and Puregene DNA extraction methods. The average relative LTL was measured using a modified singleplex quantitative polymerase chain reaction (PCR) method adapted from Cawthon (15) (50). All samples were measured in triplicates, and the average relative LTL was calculated using qBasePlus 2.0 software and expressed as a calibrated normalized relative quantity. PCR efficiency was evaluated with standard curves, and the average efficiencies were 103% for T reactions and 95% for S reactions, with *R*^2^ > 0.99 for all standard curves. The intraclass correlation coefficient for triplicate T/S ratios was 0.977 (95% CI, 0.976 to 0.978; *p* < .0001), with a coefficient of variation of 5.26%. A more detailed description can be found in the Supplement (Supplementary 1).

### Statistical Analysis

**Figure 3:**
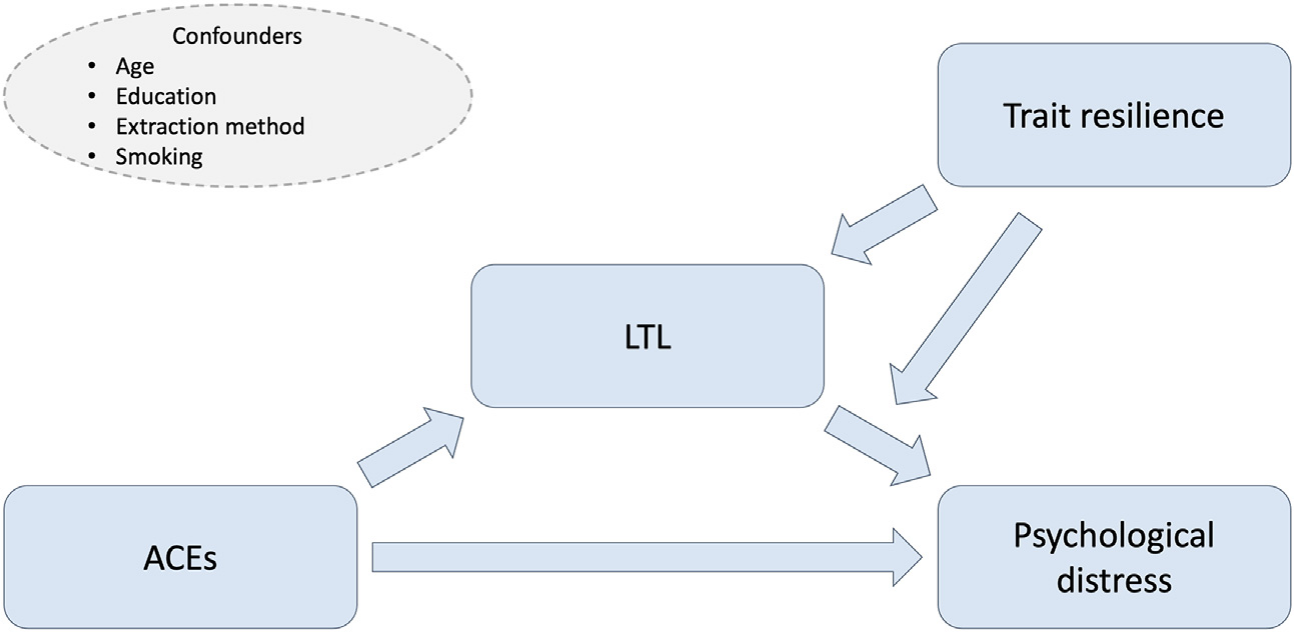
Investigated associations of the main variables and the confounders in the analyses. ACEs refer to the Trauma and Distress Scale score, LTL refers to the leukocyte telomere length, distress refers to either the Edinburgh Postnatal Depression Scale or Symptom Checklist-90 scores, and trait resilience refers to the Connor-Davidson Resilience Scale 10 score. ACE, adverse childhood experience.

Figure 3 presents an overview of the associations investigated. Analyses were performed separately for mothers and fathers. The rationale for this separate analysis is provided in Supplementary 2. *p* Values (2-tailed) < .05 were considered statistically significant. Beta coefficients (*b*), marginal *R*^2^, 95% CIs, and 95% bootstrap (BS) CIs were calculated because the distribution of models’ residuals were skewed. Bias-corrected and accelerated (BCa) CIs (based on 1000 bootstrap samples) were used for standard linear models, while percentile CIs (based on 1000 bootstrap samples) were used for mixed-effects models. Regression analyses were performed using R (version 4.2.2); the libraries boot, lme4, and ggplot2 were used (51, 52, 53, 54). The R scripts used can be found from Zenodo (https://zenodo.org/records/14808026).

#### Mediation Analysis

Mediation analysis was conducted to analyze how LTL mediates the effect of ACEs on parental distress, using the following models:

(1) M1a:distress∼intercept+ACEs+education+smoking+age

(2) M1b:LTL∼intercept+ACEs+education+smoking+age+(1|DNAextractionmethod)

where model M1a was a standard linear model, and model M1b was a linear mixed-effects model. In model M1a, the response variable distress was the average total score on the EPDS or SCL-90, calculated as the mean of the 3 total scores measured during pregnancy (gwk 14, 24, and 34). Missing values were replaced with the available total scores. Distress, ACEs, age, and LTL were used as continuous variables. Education (low, mid, high) and smoking (yes, no) were used as categorical variables. Random factor and DNA extraction method (Chemagen, Puregene) were added to models where LTL was the dependent variable because the values of LTL were not independent of the extraction method used (Supplementary 3 and Figure S1).

Based on model M1b, as the association between ACEs and LTL was not statistically significant, the mediation analysis was concluded at that point.

#### Main Effects and Moderation Analysis

First, the main effect for the association between resilience and LTL was examined using the following linear mixed-effects model:

(3) M2:LTL∼intercept+resilience+education+smoking+age+(1|DNAextractionmethod)

where all the variables were as described above. Next, the main effects of LTL and resilience were examined using the following linear model:

(4) M3:distress∼intercept+LTL+resilience+education+smoking+age

where all the variables were as described above. Finally, the moderation analysis was formulated by including an interaction term between LTL and resilience in model M3.

#### Additional Analysis

A post hoc analysis was used to examine the association between each ACE subscale and LTL using model M1b, replacing the total ACE score with the ACE subscales. ACE subscales were treated as categorical variables (Table 2) and as a continuous variable (Table S1). Furthermore, we investigated the association between selected covariates (LTL storage time, maternal body mass index at the beginning of pregnancy, maternal and paternal age at the child’s birth, multiparity) and LTL using a mixed-effects model, with extraction method as a random factor (Table S2). As a sensitivity analysis, nonlinear associations between LTL and age, ACEs, resilience, and distress were examined using a piecewise linear function, i.e., spline, allowing for breaks at the lower and upper quartiles, as well as the median values for each variable (Tables S3 and S4). Additionally, a sensitivity analysis of participants who did not report taking selective serotonin reuptake inhibitors or corticosteroid medication at gwk 14 showed qualitatively similar results (Tables S4–S7). Because these additional analyses did not produce qualitatively different results, detailed results are reported only in the Supplement.

**Table 2:** Results of the Mediation Model Illustrating How LTL Mediates the Effect of ACEs on Depressive and Anxiety Symptoms, With Additional Results Showing the Mediation Effect Within the ACE Subscales

Outcome	Mothers				Fathers			
	*b* (95% CI)	95% BS CI	*p*	*R*^2^	*b* (95% CI)	95% BS CI	*p*	*R*^2^
Depressive Symptoms								
(Intercept)	5.47 (2.74 to 8.19)	2.86 to 8.22	<.001	–	2.28 (0.63 to 3.93)	0.64 to 3.88	.007	–
ACEs	0.09 (0.06 to 0.12)	0.05 to 0.13	<.001	0.097	0.10 (0.07 to 0.13)	0.06 to 0.14	<.001	0.107
Education, Mid^a^	−0.36 (−1.31 to 0.59)	−1.33 to 0.56	.460	0.002	−0.11 (−0.81 to 0.59)	−0.76 to 0.55	.760	<0.001
Education, High^a^	−0.17 (−1.10 to 0.76)	−1.08 to 0.77	.716	<0.001	0.28 (−0.45 to 1.02)	−0.47 to 1.13	.445	0.002
Smoking, No^b^	−0.34 (−1.59 to 0.90)	−1.82 to 1.08	.587	0.001	0.12 (−0.52 to 0.77)	−0.59 to 0.75	.707	<0.001
Age	−0.04 (−0.12 to 0.05)	−0.12 to 0.05	.409	0.002	−0.01 (−0.06 to 0.04)	−0.06 to 0.04	.727	<0.001

Anxiety Symptoms								
(Intercept)	3.86 (1.14 to 6.57)	1.42 to 6.50	.006	–	1.91 (0.22 to 3.59)	0.61 to 3.27	.027	–
ACEs	0.11 (0.08 to 0.14)	0.07 to 0.14	<.001	0.131	0.09 (0.05 to 0.12)	0.04 to 0.14	<.001	0.078
Education, Mid^a^	−0.63 (−1.58 to 0.31)	−1.67 to 0.37	.190	0.005	0.02 (−0.69 to 0.73)	−0.68 to 0.64	.956	<0.001
Education, High^a^	−0.74 (−1.66 to 0.19)	−1.76 to 0.12	.119	0.008	0.54 (−0.21 to 1.29)	−0.24 to 1.53	.155	0.006
Smoking, No^b^	0.27 (−0.97 to 1.52)	−1.14 to 1.54	.664	0.001	−0.13 (−0.79 to 0.53)	−0.88 to 0.56	.694	<0.001
Age	−0.04 (−0.13 to 0.05)	−0.12 to 0.04	.350	0.003	−0.02 (−0.07 to 0.03)	−0.06 to 0.03	.390	0.002
LTL								
(Intercept)	1.05 (0.80 to 1.30)	0.80 to 1.29	<.001	–	0.99 (0.84 to 1.14)	0.83 to 1.13	<.001	–
ACEs	0.00 (0.00 to 0.00)	0.00 to 0.00	.730	<0.001	0.00 (0.00 to 0.00)	0.00 to 0.00	.715	<0.001
Education, Mid^a^	0.05 (−0.02 to 0.11)	−0.03 to 0.12	.194	0.004	0.01 (−0.06 to 0.07)	−0.06 to 0.07	.871	<0.001
Education, High^a^	0.01 (−0.05 to 0.08)	−0.05 to 0.08	.687	<0.001	0.02 (−0.04 to 0.09)	−0.04 to 0.09	.478	0.002
Smoking, No^b^	−0.01 (−0.10 to 0.08)	−0.10 to 0.09	.880	<0.001	0.05 (−0.01 to 0.10)	−0.01 to 0.11	.122	0.008
Age	−0.01 (−0.01 to 0.00)	−0.01 to 0.00	.087	0.008	0.00 (−0.01 to 0.00)	−0.01 to 0.00	.033	0.014
ACE Subscales^c^								
Emotional neglect, 1 event	0.14 (0.06 to 0.23)	0.06 to 0.23	<.001	0.028	−0.02 (−0.12 to 0.08)	−0.12 to 0.08	.697	<0.001
Emotional neglect, 2 or more events	0.03 (−0.03 to 0.10)	−0.03 to 0.10	.328	0.002	0.03 (−0.05 to 0.10)	−0.04 to 0.10	.506	0.001
Emotional abuse, 1 event	−0.06 (−0.14 to 0.02)	−0.14 to 0.02	.144	0.006	0.01 (−0.06 to 0.08)	−0.06 to 0.08	.811	<0.001
Emotional abuse, 2 or more events	−0.03 (−0.09 to 0.03)	−0.09 to 0.03	.302	0.003	−0.03 (−0.09 to 0.03)	−0.09 to 0.03	.313	0.003
Physical neglect, 1 event	0.00 (−0.08 to 0.07)	−0.07 to 0.07	.921	<0.001	−0.07 (−0.16 to 0.01)	−0.16 to 0.01	.092	0.009
Physical neglect, 2 or more events	0.02 (−0.04 to 0.08)	−0.04 to 0.08	.576	0.001	−0.04 (−0.11 to 0.03)	−0.10 to 0.03	.316	0.003
Physical abuse, 1 event	−0.02 (−0.09 to 0.06)	−0.09 to 0.07	.703	<0.001	0.04 (−0.03 to 0.11)	−0.03 to 0.11	.277	0.004
Physical abuse, 2 or more events	−0.01 (−0.07 to 0.05)	−0.07 to 0.05	.761	<0.001	−0.01 (−0.07 to 0.05)	−0.07 to 0.05	.706	<0.001
Sexual abuse, 1 event	−0.08 (−0.20 to 0.04)	−0.20 to 0.04	.178	0.005	0.02 (−0.15 to 0.19)	−0.15 to 0.18	.845	<0.001
Sexual abuse, 2 or more events	0.07 (−0.02 to 0.15)	−0.02 to 0.14	.149	0.005	−0.11 (−0.29 to 0.07)	−0.3 to 0.07	.220	0.005

ACE, adverse childhood experience; BS, bootstrap; LTL, leukocyte telomere length.

a The reference level is low.

b The reference level is yes.

c Only the estimates for ACE subscales are shown. Each model was also adjusted for education, smoking, and age.

## Results

### Resilience, ACEs, and Psychological Distress Characteristics

The mean scores for trait resilience, ACEs, and depressive and anxiety symptoms are presented in Table 1. The most common ACEs were emotional and physical neglect; 78% of mothers and 85% of fathers reported emotional neglect, and 72% and 81% reported physical neglect, respectively. Emotional abuse was reported by 59% of mothers and 53% of fathers; physical abuse was reported by 45% of mothers and 51% of fathers; and sexual abuse was reported by 15% of mothers and 5% of fathers.

### The Associations and Mediation Effect of LTL With Psychological Distress and ACEs

The findings did not indicate a significant association between LTL and depressive symptoms for mothers (*b* = 0.81; 95% BCa CI, −0.43 to 2.33; *p* = .27) or fathers (*b* = −0.34; 95% BCa CI, −1.53 to 1.17; *p* = .56) or between LTL and anxiety symptoms (mothers: *b* = 0.92; 95% BCa CI, −0.47 to 2.32; *p* = .24; fathers: *b* = −0.62; 95% BCa CI, −1.74 to 1.21; *p* = .32). The results indicated a significant association between ACEs and depressive symptoms (mothers: *b* = 0.09; 95% BCa CI, 0.05 to 0.13; *p* < .001; fathers: *b* = 0.10; 95% BCa CI, 0.06 to 0.14; *p* < .001) and anxiety symptoms (mothers: *b* = 0.11; 95% BCa CI, 0.08 to 0.14; *p* < .001; fathers: *b* = 0.09; 95% BCa CI, 0.04 to 0.14; *p* < .001). However, shorter LTL was not found to be associated with ACEs and, therefore, could not mediate the hypothesized association between ACEs and psychological distress symptoms (mothers: *b* = 0.00; 95% BCa CI, 0.00 to 0.00; *p* = .73; fathers: *b* = 0.00; 95% BCa CI, 0.00 to 0.00; *p* = .72). The ACE subscales were explored by comparing individuals with ≥1 ACE to individuals with no ACEs within each subscale. One significant finding emerged among mothers: mothers who reported 1 score indicating emotional neglect had longer telomeres than mothers who reported no emotional neglect experiences (*b* = 0.14; 95% BCa CI, 0.06 to 0.23; *p* < .001). The results of the mediation models are presented in Table 2.

### The Associations Between Trait Resilience and LTL

**Figure 4:**
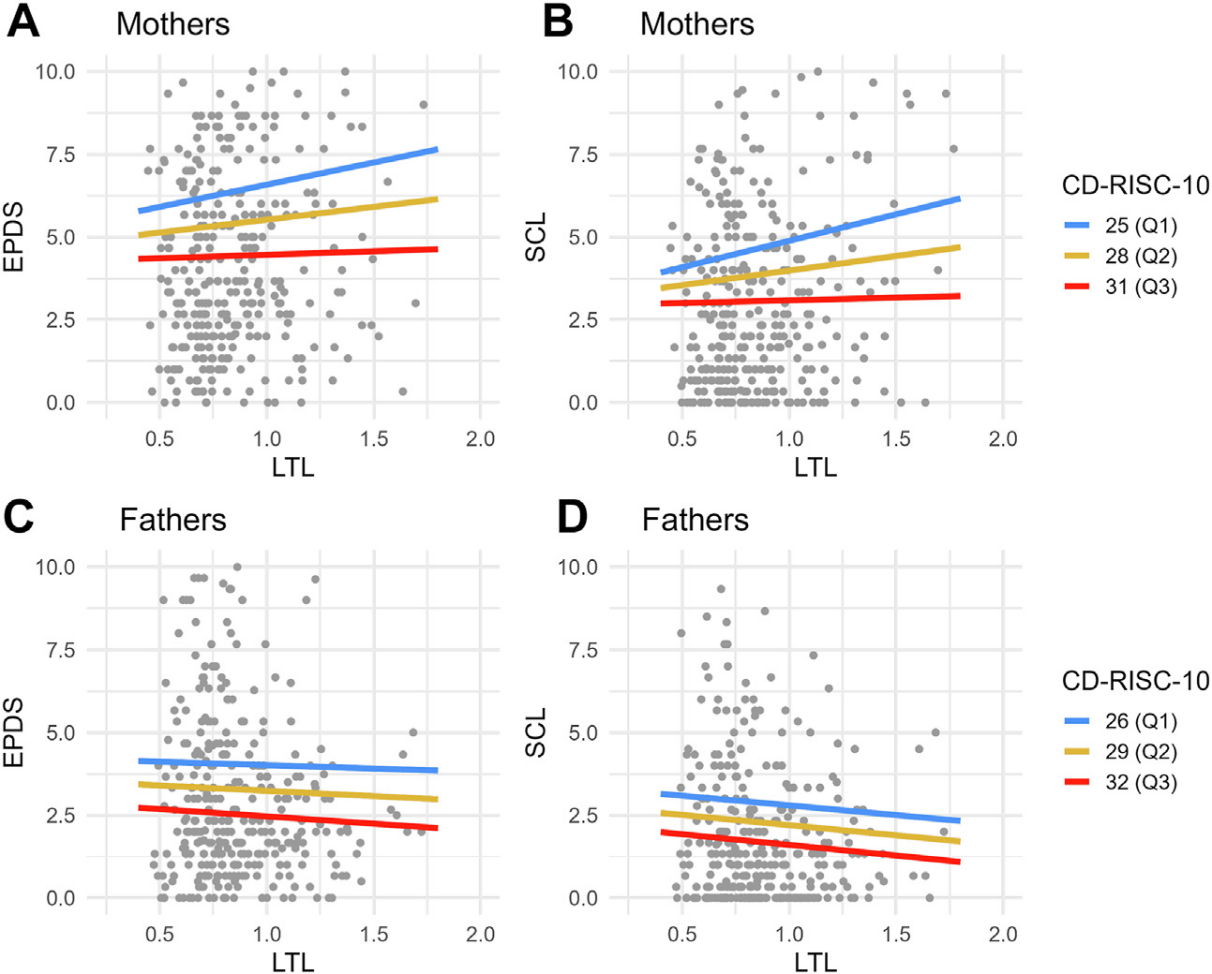
Moderation model examining the interactions between trait resilience, LTL, and depressive and anxiety symptoms. The CD-RISC-10 was used as a continuous variable in the statistical analyses, and for illustrative purposes, the quartiles Q1, Q2, and Q3 were estimated. To enhance clarity, the y-axis has been truncated at 10 points. CD-RISC-10, Connor-Davidson Resilience Scale 10; EPDS, Edinburgh Postnatal Depression Scale; LTL, leukocyte telomere length; SCL, Symptom Checklist-90.

The relationship between trait resilience and LTL yielded no significant findings for mothers (*b* = 0.00; 95% BS CI, −0.01 to 0.00; *p* = .32) or fathers (*b* = 0.00; 95% BS CI, −0.01 to 0.00; *p* = .31). The associations between trait resilience and LTL are presented in Table 3. Furthermore, no moderating effect of trait resilience was observed either for the relationship between LTL and depressive symptoms (mothers: *b* = −0.19; 95% BCa CI, −0.46 to 0.12; *p* = .27; fathers: *b* = −0.04; 95% BCa CI, −0.42 to 0.33; *p* = .76) or for the relationship between LTL and anxiety symptoms (mothers: *b* = −0.24; 95% BCa CI, −0.53 to 0.10; *p* = .17; fathers: *b* = −0.01; 95% BCa CI, −0.51 to 0.31; *p* = .95). Additional details are provided in Table 4 and Figure 4.

**Table 3:** Results From a Linear Mixed-Effects Model Investigating the Associations of LTL With Trait Resilience, Education, Age, and Smoking

	Mothers				Fathers			
	*b* (95% CI)	95% BS CI	*p*	*R*^2^	*b* (95% CI)	95% BS CI	*p*	*R*^2^
(Intercept)	1.12 (0.84 to 1.41)	0.83 to 1.39	<.001	–	0.90 (0.68 to 1.12)	0.68 to 1.11	<.001	–
Trait Resilience	0.00 (−0.01 to 0.00)	−0.01 to 0.00	.320	0.003	0.00 (0.00 to 0.01)	0.00 to 0.01	.310	0.003
Education, Mid^a^	0.05 (−0.02 to 0.12)	−0.02 to 0.12	.155	0.005	0.01 (−0.06 to 0.07)	−0.06 to 0.06	.861	<0.001
Education, High^a^	0.02 (−0.05 to 0.09)	−0.05 to 0.08	.576	0.001	0.02 (−0.04 to 0.09)	−0.04 to 0.09	.492	0.001
Age	−0.01 (−0.01 to 0.00)	−0.01 to 0.00	.109	0.007	0.00 (−0.01 to 0.00)	−0.01 to 0.00	.036	0.014
Smoking, No^b^	−0.01 (−0.10 to 0.08)	−0.11 to 0.07	.829	<0.001	0.05 (−0.01 to 0.11)	−0.01 to 0.11	.104	0.008

BS, bootstrap; LTL, leukocyte telomere length.

a The reference level is low.

b The reference level is yes.

**Table 4:** Results of the Moderation Models of Trait Resilience, LTL, and Depressive and Anxiety Symptoms, With Interaction Analysis Between Trait Resilience and LTL

Outcome	Mothers				Fathers			
	*b* (95% CI)	95% BS CI	*p*	*R*^2^	*b* (95% CI)	95% BS CI	*p*	*R*^2^
Depressive Symptoms								
Main Effects								
(Intercept)	13.17 (9.56 to 16.78)	9.59 to 16.56	<.001	–	11.23 (8.71 to 13.74)	8.60 to 14.47	<.001	–
LTL	0.81 (−0.64 to 2.25)	−0.43 to 2.33	.273	0.004	−0.34 (−1.50 to 0.81)	−1.53 to 1.17	.559	0.001
Trait resilience	−0.32 (−0.40 to −0.24)	−0.40 to −0.24	<.001	0.167	−0.25 (−0.30 to −0.20)	−0.32 to −0.18	<.001	0.212
Education, mid^a^	0.12 (−0.80 to 1.04)	−0.74 to 1.11	.795	<0.001	−0.21 (−0.87 to 0.44)	−0.85 to 0.43	.521	0.001
Education, high^a^	0.22 (−0.69 to 1.12)	−0.68 to 1.14	.639	0.001	0.31 (−0.37 to 1.00)	−0.39 to 1.03	.369	0.003
Age	0.02 (−0.07 to 0.10)	−0.07 to 0.12	.662	0.001	−0.01 (−0.06 to 0.03)	−0.06 to 0.04	.614	0.001
Smoking, no^b^	−0.77 (−1.97 to 0.43)	−2.20 to 0.51	.206	0.005	−0.17 (−0.77 to 0.44)	−0.83 to 0.41	.588	0.001
Moderation								
LTL × trait resilience	−0.19 (−0.51 to 0.14)	−0.46 to 0.12	.256	0.004	−0.04 (−0.29 to 0.21)	−0.42 to 0.33	.755	<0.001

Anxiety Symptoms								
Main effects								
(Intercept)	9.99 (6.19 to 13.79)	6.34 to 13.85	<.001		9.34 (6.68 to 12.00)	6.42 to 12.68	<.001	
LTL	0.92 (−0.60 to 2.44)	−0.47 to 2.32	.235	0.004	−0.62 (−1.84 to 0.60)	−1.74 to 1.21	.318	0.003
Trait resilience	−0.26 (−0.34 to −0.17)	−0.34 to −0.17	<.001	0.105	−0.20 (−0.25 to −0.14)	−0.27 to −0.13	<.001	0.129
Education, mid^a^	−0.26 (−1.23 to 0.71)	−1.28 to 0.75	.597	0.001	−0.07 (−0.76 to 0.62)	−0.73 to 0.56	.839	<0.001
Education, high^a^	−0.52 (−1.47 to 0.44)	−1.50 to 0.33	.287	0.004	0.56 (−0.16 to 1.29)	−0.18 to 1.55	.127	0.007
Age	0.01 (−0.08 to 0.10)	−0.07 to 0.10	.780	<0.001	−0.03 (−0.07 to 0.02)	−0.07 to 0.01	.318	0.003
Smoking, no^b^	−0.11 (−1.37 to 1.15)	−1.48 to 1.08	.865	<0.001	−0.36 (−1.00 to 0.27)	−1.04 to 0.37	.262	0.004
Moderation								
LTL × trait resilience	−0.24 (−0.58 to 0.10)	−0.53 to 0.10	.171	0.006	−0.01 (−0.27 to 0.25)	−0.51 to 0.31	.945	<0.001

The table only displays estimates for the interaction term in the moderation analysis. The moderation analysis included the same variables as the main effects model.

BS, bootstrap; LTL, leukocyte telomere length.

a The reference level is low.

b The reference level is yes.

### Age and LTL

Overall, both maternal and paternal age exhibited a tendency toward telomere shortening with increasing age, as indicated by visual inspection of the data. In addition, the correlation between LTL and age was significant in mothers (*r* = −0.08, *p* < .05). However, when the extraction method was controlled for, the association between age and LTL was no longer significant (mothers: *b* = −0.006; 95% CI, −0.011 to 0.001; *p* = .068; fathers: *b* = −0.004; 95% CI, −0.008 to 0.003; *p* = .074).

### Correlations Among All Variables

To provide further context, Table S8 presents correlations among all the investigated variables, including main variables and covariates. Most covariates exhibited minimal associations with LTL. Age and smoking showed potential associations with LTL, but no other variables demonstrated notable correlations. Likewise, no covariates significantly predicted LTL in our regression models. However, LTL was clearly associated with the DNA extraction method, as shown in the boxplot (Figure S1).

## Discussion

The objective of this study was to examine the potential associations between LTL, trait resilience, depressive and anxiety symptoms, and ACEs. Previous research has consistently shown links between shorter LTL, psychiatric disorders, and psychosocial stress (16,17). Nevertheless, in contrast to our first hypothesis, our findings did not reveal any associations between depressive or anxiety symptoms and LTL. Moreover, although previous research has demonstrated an association between ACEs and shorter LTL (18, 19, 20), our study did not support this, in contrast to our second hypothesis. One significant result emerged among mothers, where moderate emotional neglect was associated with longer telomeres. However, this association did not persist when emotional neglect was treated as a continuous variable. No significant associations were found for the total ACE score or any other ACE subscale. Given the lack of a consistent pattern and the multiple comparisons conducted, this finding should be interpreted with caution and may be due to chance. Our third hypothesis postulated that LTL may mediate the effect of ACEs on distress symptoms. However, because no association was found between ACEs and LTL, no mediation effect could be observed. The discrepancy between our findings and the findings of the previous studies may stem from differences in sample characteristics. Previous studies often focused on populations with more severe symptoms and clinical disorders, whereas the participants in our sample exhibited relatively low frequencies of ACEs and generally mild levels of depression and anxiety. This may partially explain the lack of observed association in our study, because evidence suggests that the cumulative impact of ACEs may play a role (20). Although there is convincing evidence that links psychological symptoms, ACEs, and shorter telomeres, conflicting findings, such as those of the current study, also exist. A recent study that examined TL in individuals with and without major depression and ACEs found no associations (28). One potential explanation that they suggested for their null findings was the comparatively good physical health of their participants (28).

Given the association between trait resilience and better mental health (2,3) and the potential protection provided by trait resilience against the negative impact of ACEs (4, 5, 6), in this study, we aimed to investigate the potential associations between trait resilience and LTL. Few studies have investigated the relationship between TL and resilience, defined more broadly than our trait resilience definition, with findings suggesting that higher resilience is associated with longer TL. However, we did not find any significant association between trait resilience and LTL in our study, in contrast to our initial hypothesis. Our final hypothesis proposed that trait resilience might moderate the relationship of LTL and distress symptoms, but this was not confirmed. The existing research on resilience and telomeres has primarily focused on an older population compared with this study, which together with differing definitions of resilience may explain the contrasting findings.

Our findings showing that ACEs were associated with depressive and anxiety symptoms are consistent with the findings of numerous other studies (22, 23, 24). While it has been proposed that the adverse effects of ACEs may be mediated by shortened TL (25,26), this hypothesis was not supported in the current study. This outcome could suggest that adverse experiences encountered during childhood do not necessarily lead to telomere shortening. In fact, other factors, such as poorer health behaviors, may contribute to telomere attrition (55,56). ACEs have been associated with alcohol use, smoking, poor diet, and obesity (57, 58, 59, 60, 61), which may mediate the impact of ACEs on telomeres. In the current study, the participants generally represent a population with good socioeconomic status, which can facilitate healthier behaviors and even reduce chronic stress, potentially partially explaining the contrasting findings compared with previous studies.

### Limitations and Future Directions

This study has limitations that should be considered when interpreting the results. The predominantly White study population may limit the generalizability of the results. A multiethnic study found no overall relationship between depression and TL, although a significant association was observed in non-Hispanic White participants (62). Our sample of expectant mothers should also be considered, because pregnancy may influence telomeres, although the evidence remains inconclusive (63,64). Notwithstanding its limitations, this study is one of a small number of studies that have examined resilience and LTL. To the best of our knowledge, it is the first study to explore their relationship during pregnancy. It is also noteworthy that this study examined both mothers and fathers, with fathers having been the subject of significantly less research. Future studies should examine resilience in a more diverse population, including participants of various ethnic backgrounds. It would also be valuable to consider lifestyle factors, such as physical activity and eating habits.

### Conclusions

Previous studies have reported associations between shorter TL, ACEs, and psychological distress symptoms. However, conflicting findings have also been documented. The current study did not yield evidence for such associations. We also sought to examine a potential correlation between trait resilience and LTL but found no significant relationship. The lack of significant associations in our study may be attributed to several factors, particularly the characteristics of our study population. The prevalence of ACEs was relatively low; depressive and anxiety symptoms were mild on average; and participants generally represented a higher socioeconomic status, all of which can influence both mental and physical health outcomes.

To the best of our knowledge, no previous studies have explored the relationship between self-reported trait resilience and TL. Although no significant associations with LTL were identified, it is plausible that the effects of trait resilience may be more pronounced in populations with higher levels of distress or a greater burden of ACEs. Notably, we observed an association between ACEs and depressive and anxiety symptoms, with more adverse experiences being correlated with higher levels of distress. In this context, might it not be seen as a positive outcome if adverse experiences themselves do not directly lead to shorter telomeres? Instead, other factors, such as lifestyle behaviors, may mediate the negative consequences of ACEs. This perspective is encouraging because it suggests that interventions that target these mediating factors may help counteract the negative effects of adverse experiences.

## Supplementary Material

Supplemental Text, Figures S1–S2, and Tables S1–S8

Key Resources Table
