# Supplementary material for: Associations of Leukocyte Telomere Length With Trait Resilience, Adverse Childhood Experiences, and Psychological Distress Among Expecting Parents in the FinnBrain Birth Cohort
Source: Biol Psychiatry Glob Open Sci. 2025 Apr 1;5(4):100498. doi: 10.1016/j.bpsgos.2025.100498 (PMC12142313; doi:10.1016/j.bpsgos.2025.100498)
Supplement: Supplemental Text, Figures S1–S2, and Tables S1–S8 [file mmc1.pdf]

SUPPLEMENTARY INFORMATION

Associations of Leukocyte Telomere Length With Trait Resilience, Adverse Childhood Experiences, and Psychological Distress Among Expecting Parents in the FinnBrain Birth Cohort

Mondolin *et al.*

Supplementary 1: DNA Extraction and Leukocyte Telomere Length

DNA was extracted at the DNA unit of the National Institute for Health and Welfare in accordance with the standard protocol using Chemagen and Puregene DNA extraction methods. The average relative telomere length was measured using a modified singleplex quantitative PCR (qPCR) method adapted from Cawthon, 2002 and 2009 (1,2). To ensure a uniform DNA input of 5 ng for each qPCR reaction, samples were diluted and checked using the Qubit™ dsDNA High Sensitivity Assay Kit (Life Technologies, Europe) using the Qubit™ Flex Fluorometer (Life Technologies, Europe). All samples were measured in triplicate on a QuantStudio 5 real-time PCR system (Applied Biosystems) in a 384-well format.

First, a single copy gene (S) reaction (human β globin) was performed. This reaction mixture contained 5 ng DNA template, 1× KAPA SYBR® FAST, Low ROX™ master mix (Kapa Biosystems, Merck), 450 nM HBG1 primer (GCTTCTGACACAACTGTGTTCACTAGC), and 450 nM HBG2 primer (CACCAACTTCATCCACGTTCACC). Cycling conditions were as follows: 1 cycle at 95°C for 3 min, 40 cycles at 95°C for 3 sec, and 58°C for 15 sec.

Second a telomere-specific (T) reaction was performed. This reaction mixture contained 5 ng DNA template, 1× KAPA SYBR® FAST, Low ROX™ master mix (Kapa Biosystems. Merck), 2 mM DTT, 100 nM TelG primer (ACACTAAGGTTTGGGTTTGGGTTTGGGTTTGGGTTAGTGT), and 100 nM TelC primer (TGTTAGGTATCCCTATCCCTATCCCTATCCCTATCCCTAACA). Cycling conditions were as follows: 1 cycle at 95°C for 3 min, 2 cycles at 94°C for 3 sec and 49°C for 15 sec, and 30 cycles at 94°C for 3 sec, 62°C for 5 sec, and 74°C for 10 sec.

After each qPCR, a melting curve analysis was performed, and the qPCR curves were visually inspected. Samples with technical issues were excluded. For each triplicate, Cq values from the S and T runs were examined, with those with deviations exceeding 0.3 being excluded. On each run, PCR efficiency was evaluated using two 6-point serially diluted standard curves (efficiencies were on average 103% for T reactions, and 95% for S reactions, with $R^2 > 0.99$ for all standard curves).

The average relative LTL was calculated using qBasePlus 2.0 software (Biogazelle) and expressed as a calibrated normalized relative quantity (CNRQ). The latter is achieved by first calculating RQ based on the delta-Cq method for T and S Cq values. As the choice of a calibrator sample (sample to which subsequent normalization is performed, i.e., delta-delta-Cq) strongly influences the error on the final relative quantities due to measurement error on the calibrator sample, normalization is performed to arithmetic mean quantification values for all analyzed samples, resulting in the NRQ.

As samples are measured over different qPCR plates, we ran sixteen inter-run calibrator samples (IRCs) on every plate and used them to calculate an additional calibration factor to eliminate run-to-run differences, resulting in the final T/S ratio (CNRQ). Mathematical calculation formulas to obtain RQ, NRQ, and CNRQs are provided by Hellemans et al. 2007 (3).

The intraclass correlation coefficient (ICC) of the triplicate T/S ratios was 0.977 (95% CI: 0.976 to 0.978; p < .0001), with a coefficient of variation (CV) of 5.26%. For the sixteen IRCs that were measured on each qPCR plate, the ICC was 0.984 (95% CI: 0.963 to 0.991; p < .0001) with a CV of 5.05%.

Supplementary 2: Rationale for Separate Analyses of Mothers and Fathers

In this study, we opted to analyze mothers and fathers separately, as pregnant mothers undergo distinct physiological and hormonal changes that can affect both telomere length and psychological distress in ways not applicable to fathers. Separate analyses help ensure that potential group differences are accurately identified rather than obscured in a combined analysis.

Supplementary 3: Extraction Methods as a Confounding Factor

Two different DNA extraction methods, Chemagen and Puregene, were used in this study. As LTL values appeared to be influenced by the extraction method, this factor was accounted for as a confounder in the analyses. Notably, the Puregene method yielded higher LTL values than the Chemagen method (mean [SD]: 0.935 [0.23] vs. 0.848 [0.24], $p < 0.01$). Moreover, the sample sizes differed substantially between the two methods ($n = 64$ vs. $n = 630$), further underscoring the importance of controlling for extraction method to ensure the robustness of the findings. To illustrate these statistically significant differences in LTL values, a boxplot is provided (Supplementary Figure 1).

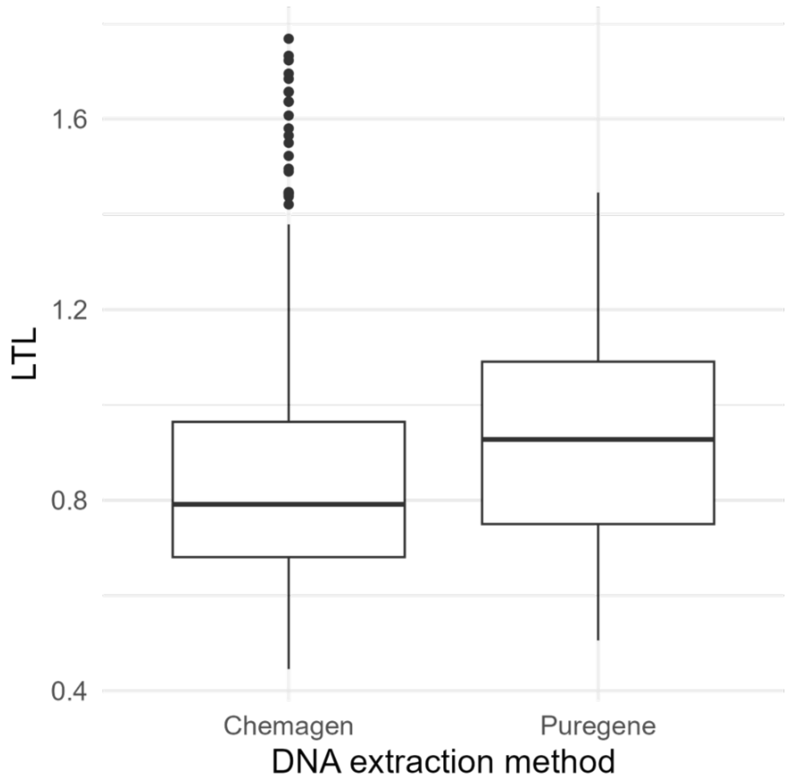

Supplementary Figure 1. Association between leukocyte telomere length (LTL) and DNA extraction method, illustrated using a boxplot.

Supplementary Table 1 Results from the mixed-effects model on the associations between LTL and ACEs subscales modelled as continuous.

		Mothers				Fathers			
Outcome		b (95% CI)	95% BS CI	*p*	R^2	b (95% CI)	95% BS CI	*p*	R^2
LTL	ACEs subscales[a]								
	Emotional neglect	0.00 (-0.01;0.01)	-0.01;0.01	.839	<0.001	0.00 (-0.01;0.01)	-0.01;0.01	.738	<0.001
	Emotional abuse	0.00 (-0.01;0.01)	-0.01;0.01	.603	0.001	0.00 (-0.01;0.01)	-0.01;0.01	.972	<0.001
	Physical neglect	0.00 (-0.01;0.01)	-0.01;0.01	.649	0.001	0.00 (-0.01;0.01)	-0.01;0.01	.655	0.001
	Physical abuse	-0.00 (-0.01;0.01)	-0.01;0.01	.507	0.001	0.00 (-0.01;0.01)	-0.01;0.01	.747	<0.001
	Sexual abuse	-0.00 (-0.01;0.01)	-0.01;0.02	.725	<0.001	-0.03 (-0.09;0.03)	-0.09;0.03	.277	0.004

Note: [a]Only the estimates for ACEs subscales are shown. Each model was also adjusted with education, smoking and age.

Supplementary Table 2 Results from a linear mixed-effects model investigating the associations of telomere length with multiparity, maternal BMI in the beginning of pregnancy, age and storage time.

		Mothers			Fathers		
Outcome		b	95% CI	p	b	95% CI	p
LTL	(Intercept)	0.9	0.75;1.05	<.001	NA	NA	NA
	multiparity (multipara)[a]	0	-0.05;0.06	0.924	NA	NA	NA
	(Intercept)	1.02	0.53;1.51	<.001	NA	NA	NA
	BMI (log-transformed)	-0.03	-0.18;0.11	0.65	NA	NA	NA
	(Intercept)	1.08	0.84;1.31	<.001	0.99	0.85;1.14	<.001
	Age	-0.01	-0.01;0.00	0.068	0	-0.01;0.00	0.074
	(Intercept)	0.87	0.69;1.05	<.001	0.88	0.81;0.96	<.001
	Storage time 1	0.01	-0.01;0.03	0.328	0	-0.02;0.01	0.663
	(Intercept)	0.82	0.37;1.27	<.001	0.66	0.24;1.07	0.002
	Storage time 2	0	0.00;0.00	0.676	0	0.00;0.00	0.323

Note: [a]The reference level is nullipara. Storage time 1 was calculated as the differences between DNA extraction data and sample arrival date. Storage time 2 was calculated as the differences between dilute date and sample arrival date.

Supplementary Table 3 Results from a piecewise linear mixed-effects model investigating the non-linear associations of telomere length with age, ACEs, trait resilience, EPDS and SCL.

	Mothers			Fathers		
	b	95% CI	p	b	95% CI	p
(Intercept)	1.2	0.57;1.83	<.001	1.4	0.77;2.03	<.001
Age Terms:						
min – Q1	-0.01	-0.03;0.01	0.374	-0.02	-0.04;0.00	0.099
Q1 – Q2	0	-0.02;0.02	0.989	0.03	0.00;0.05	0.051
Q2 – Q3	0.01	-0.04;0.06	0.599	-0.03	-0.07;0.00	0.028
Q3 – max	-0.02	-0.04;0.00	0.065	0	-0.01;0.01	0.799
(Intercept)	1.04	0.79;1.30	<.001	0.96	0.79;1.14	<.001
ACEs Terms:						
min – Q1	0.02	-0.03;0.08	0.36	0.01	-0.02;0.04	0.701
Q1 – Q2	-0.01	-0.03;0.01	0.404	0	-0.03;0.03	0.971
Q2 – Q3	0	-0.01;0.01	0.779	0	-0.02;0.03	0.897
Q3 – max	0	-0.01;0.00	0.468	0	-0.01;0.00	0.508
Education (mid)[a]	0.04	-0.03;0.11	0.258	0.01	-0.06;0.07	0.853
Education (high)[a]	0.01	-0.05;0.08	0.695	0.03	-0.04;0.09	0.457
Age	-0.01	-0.01;0.00	0.082	0	-0.01;0.000	0.038
Smoking (no)[b]	-0.01	-0.10;0.08	0.853	0.05	-0.01;0.11	0.12

(Intercept)	0.8	0.31;1.30	0.002	0.36	-0.07;0.78	0.1
CD-RISC-10 Terms:						
min – Q1	0.01	-0.01;0.03	0.249	0.02	0.01;0.04	0.004
Q1 – Q2	-0.02	-0.06;0.01	0.144	-0.01	-0.04;0.02	0.516
Q2 – Q3	0.01	-0.02;0.04	0.486	0.02	-0.01;0.04	0.289
Q3 – max	-0.01	-0.03;0.01	0.271	-0.02	-0.04;0.00	0.019
Education (mid)[a]	0.05	-0.02;0.12	0.156	0	-0.06;0.07	0.922
Education (high)[a]	0.02	-0.05;0.08	0.651	0.03	-0.04;0.09	0.428
Age	0	-0.01;0.00	0.134	0	-0.01;0.00	0.07
Smoking (no)[b]	0	-0.09;0.09	0.936	0.05	-0.01;0.11	0.096
(Intercept)	0.99	0.73;1.25	<.001	0.94	0.77;1.12	<.001
EPDS Terms:						
min – Q1	0.04	-0.02;0.10	0.212	0.03	-0.06;0.12	0.517
Q1 – Q2	-0.03	-0.10;0.04	0.428	0.05	-0.06;0.16	0.364
Q2 – Q3	0.01	-0.03;0.05	0.555	-0.04	-0.09;0.01	0.138
Q3 – max	0.01	-0.01;0.03	0.48	0	-0.02;0.02	0.701
Education (mid)[a]	0.05	-0.02;0.12	0.158	0	-0.06;0.07	0.93
Education (high)[a]	0.02	-0.05;0.08	0.613	0.02	-0.04;0.09	0.513
Age	-0.01	-0.01;0.00	0.086	0	-0.01;0.00	0.044
Smoking (no)[b]	-0.01	-0.11;0.08	0.763	0.05	-0.01;0.10	0.119
(Intercept)	1.06	0.81;1.32	<.001	0.96	0.80;1.12	<.001
SCL-90 Terms:						
min – Q1	-0.11	-0.26;0.05	0.183	0.06	-0.21;0.33	0.648
Q1 – Q2	0.03	-0.02;0.09	0.255	0.04	-0.06;0.14	0.459
Q2 – Q3	0.01	-0.03;0.05	0.513	-0.05	-0.11;0.01	0.091
Q3 – max	0	-0.02;0.01	0.806	0	-0.02;0.01	0.83
Education (mid)[a]	0.05	-0.02;0.11	0.187	0.01	-0.05;0.07	0.783
Education (high)[a]	0.01	-0.05;0.08	0.668	0.03	-0.04;0.09	0.438
Age	-0.01	-0.01;0.00	0.084	0	-0.01;0.00	0.041
Smoking (no)[b]	-0.01	-0.10;0.08	0.845	0.05	-0.01;0.11	0.094

Note: [a]*The reference level is low.* [b]*The reference level is yes. The 'Terms' indicate a piecewise linear function's terms which was used to model the non-linear association between age, ACEs, CD-RISC-10, EPDS, SCL-90 and LTL. In each model the piecewise linear function was allowed break, i.e., turn at the lower quartile (Q1), median (Q2) and upper quartile (Q3). For mothers the quartiles were as follows: age (27, 31, 33), ACEs (2, 7, 15), CD-RISC-10 (25, 28, 31), EPDS (2.33, 4, 7) and SCL-90 (0.67, 2.67, 5.33). For fathers the quartiles were as follows: age (28, 32, 35), ACEs (4, 8,12), CD-RISC-10 (26, 29, 32), EPDS (1.33, 2.33, 4.33) and SCL-90 (0.33, 1.33, 3).*

Supplementary 4: The Linearity of the Association Between LTL and Trait Resilience

As we explored the associations non-linearly, we observed some results between LTL and trait resilience among fathers, as shown in Supplementary Table 3. To assess the robustness of these findings, sensitivity analyses were conducted for the statistically significant results. When we slightly adjusted the classification intervals by one point and removed several outliers (N = 14), the results were no longer significant (see Supplementary Table 4). In addition, the scatterplot (Supplementary Figure 2) supports this notion, indicating that the associations are not non-linear. For these reasons, the linear analyses used in this study can be considered appropriate.

Supplementary Table 4 Sensitivity analyses for the associations between fathers' LTL, age, and CD-RISC, with adjusted classification intervals and outlier removal.

Sensitivity analyses for statistically significant results (Fathers)			
	b	95% CI	p
(Intercept)	1,39	0.74–2.04	<.001
Age Terms:			
min – Q1	-0,02	-0.04—0.01	0,119
Q1 – 31[c]	0,03	-0.01—0.06	0,101
31 – Q3[c]	-0,02	-0.04—0.00	0,1
Q4 – max	0	-0.01—0.01	0,595
(Intercept)	0,44	-0.37—1.25	0,29
CD-RISC-10 Terms:			
min[d] – Q1	0,02	-0.01—0.05	0,184
Q1 – Q2	0	-0.04—0.04	0,909
Q2 – 31[e]	0	-0.04—0.05	0,893
31[e] – max	-0,01	-0.03—0.00	0,141
Education (mid)[a]	0,01	-0.06—0.07	0,842
Education (high)[a]	0,03	-0.04—0.10	0,452
Age	0	-0.01—0.00	0,054
Smoking (no)[b]	0,05	0.01—0.11	0,107

Note: [a]The reference level is low. [b]The reference level is yes. The 'Terms' indicate a piecewise linear function's terms which was used to model the non-linear association between age, ACEs, CD-RISC-10, EPDS, SCL-90 and LTL. Compared to Supplementary Table 2, [c]Q2 was replaced with Q2-1=31. [d]N=14 datapoints were removed as outliers. [e]Q3 was replaced with Q3-1.25=31.

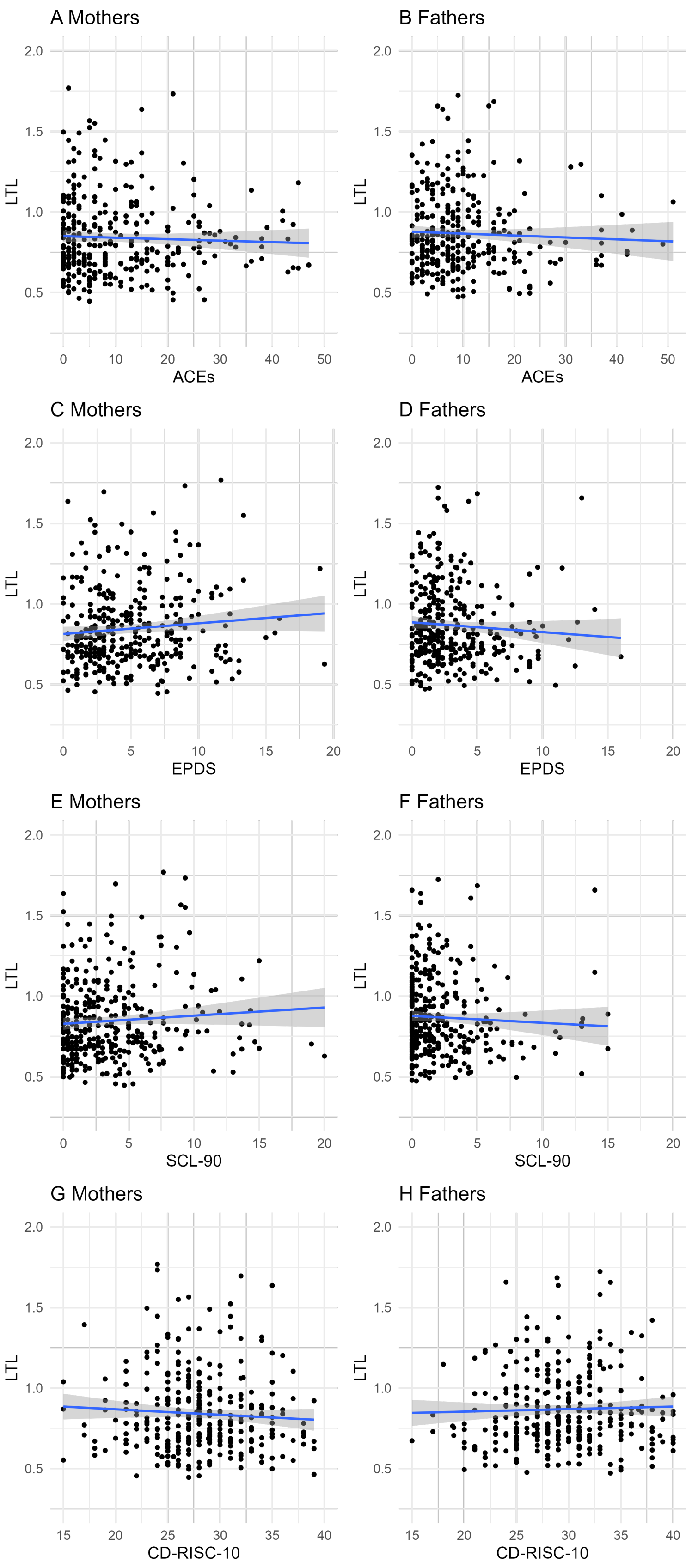

Supplementary Figure 2 Scatter plot illustrating the associations between LTL and ACEs, depressive symptoms (EPDS), anxiety symptoms (SCL), and trait resilience (CD-RISC-10).

Supplementary Table 5 Sensitivity analyses including only those who do not use medication. Results of the mediation model illustrating how LTL mediates the effect of ACEs on depressive.

		Mothers				Fathers			
Outcome		b	95% CI	95% BS CI	p	b	95% CI	95% BS CI	p
EPDS	(Intercept)	5.09	2.29;7.89	2.53;8.18	<.001	2.41	0.73;4.08	0.86;4.04	0.005
	ACEs	0.10	0.06;0.13	0.05;0.14	<.001	0.08	0.05;0.12	0.05;0.14	<.001
	Education (mid)[a]	-0.20	-1.18;0.78	-1.23;0.78	0.685	-0.19	-0.90;0.52	-0.84;0.51	0.597
	Educatio (high)[a]	0.03	-0.93;0.98	-1.00;0.92	0.957	0.22	-0.53;0.96	-0.54;1.02	0.569
	Smoking (no)[b]	-0.18	-1.46;1.11	-1.82;1.11	0.786	0.20	-0.47;0.87	-0.45;0.87	0.557
	Age	-0.04	-0.13;0.05	-0.13;0.05	0.439	-0.01	-0.06;0.04	-0.05;0.04	0.682
SCL	(Intercept)	3.96	1.22;6.71	1.56;6.92	0.005	2.06	0.36;3.75	0.82;3.56	0.018
	ACEs	0.11	0.08;0.14	0.07;0.15	<.001	0.07	0.03;0.10	0.03;0.12	<.001
	Education (mid)[a]	-0.27	-1.23;0.69	-1.32;0.63	0.578	0.04	-0.68;0.76	-0.56;0.73	0.911
	Educatio (high)[a]	-0.26	-1.20;0.67	-1.19;0.64	0.58	0.59	-0.17;1.35	-0.26;1.56	0.129
	Smoking (no)[b]	0.16	-1.10;1.41	-1.58;1.31	0.807	-0.15	-0.83;0.53	-0.90;0.51	0.662
	Age	-0.05	-0.14;0.03	-0.15;0.02	0.234	-0.02	-0.07;0.03	-0.06;0.02	0.351
LTL	(Intercept)	0.94	0.69;1.20	0.67;1.20	<.001	0.99	0.83;1.14	0.83;1.13	<.001
	ACEs	0.00	-0.00;0.00	-0.00;0.00	0.267	0.00	-0.00;0.00	-0.00;0.00	0.632
	Education (mid)[a]	0.06	-0.01;0.12	-0.02;0.12	0.104	-0.01	-0.07;0.06	-0.07;0.06	0.836
	Educatio (high)[a]	0.03	-0.04;0.09	-0.04;0.09	0.425	0.02	-0.05;0.09	-0.05;0.09	0.574
	Age	0.00	-0.01;0.00	-0.01;0.00	0.298	0.00	-0.01;0.00	-0.01;0.00	0.038
	Smoking (no)[b]	0.02	-0.07;0.11	-0.07;0.11	0.689	0.04	-0.02;0.11	-0.01;0.10	0.146

Note: [a]The reference level is low. [b]The reference level is yes.

Supplementary Table 6 Sensitivity analyses including only those who do not use medication. Results from a linear mixed-effects model investigating the associations of LTL with trait resilience, education, age, and smoking.

		Mothers				Fathers			
Outcome		b	95% CI	95% BS CI	p	b	95% CI	95% BS CI	p
LTL	(Intercept)	1.00	0.71;1.28	0.70;1.29	<.001	0.89	0.66;1.11	0.65;1.12	<.001
	CD-RISC-10	0.00	-0.01;0.00	-0.01;0.00	0.563	0.00	0.00;0.01	0.00;0.01	0.282
	Education (mid)[a]	0.06	-0.01;0.13	-0.01;0.12	0.096	-0.01	-0.07;0.06	-0.07;0.06	0.85
	Educatio (high)[a]	0.03	-0.04;0.09	-0.04;0.10	0.419	0.02	-0.05;0.09	-0.05;0.08	0.604
	Age	0.00	-0.01;0.00	-0.01;0.00	0.35	0.00	-0.01;0.00	-0.01;0.00	0.041
	Smoking (no)[b]	0.02	-0.07;0.10	-0.07;0.10	0.737	0.05	-0.01;0.11	-0.01;0.10	0.115

Note: [a]The reference level is low. [b]The reference level is yes.

Supplementary Table7 Sensitivity analyses including only those who do not use medication. Results of the moderation models of trait resilience, LTL, and depressive and anxiety symptoms, with interaction analysis between trait resilience and LTL.

		Mothers				Fathers			
Outcome		b	95% CI	95% BS CI	p	b	95% CI	95% BS CI	p
EPDS	Main effects:								
	(Intercept)	13.34	9.67;17.01	9.87;17.16	<.001	10.81	8.27;13.35	8.17;14.01	<.001
	LTL	0.54	-1.05;2.13	-0.94;2.25	0.501	-0.16	-1.34;1.02	-1.47;1.33	0.785
	CD-RISC-10	-0.32	-0.40; -0.24	-0.41; -0.23	<.001	-0.24	-0.29; -0.19	-0.32; -0.17	<.001
	Education (mid)[a]	0.22	-0.73;1.18	-0.77;1.15	0.646	-0.27	-0.93;0.39	-0.88;0.39	0.423
	Education (high)[a]	0.28	-0.65;1.21	-0.68;1.08	0.551	0.31	-0.38;1.01	-0.41;0.91	0.377
	Age	0.01	-0.07;0.10	-0.08;0.11	0.739	-0.02	-0.06;0.03	-0.06;0.03	0.524
	Smoking (no)[b]	-0.66	-1.90;0.58	-1.98;0.46	0.297	-0.12	-0.74;0.49	-0.74;0.49	0.693
	Moderation:								
	LTL x CD-RISC-10	-0.12	-0.47;0.23	-0.42;0.23	0.504	-0.08	-0.34;0.17	-0.50;0.26	0.518
SCL	Main effects:								
	(Intercept)	10.13	6.33;13.93	6.42;14.84	<.001	8.99	6.32;11.65	6.31;12.86	<.001
	LTL	0.88	-0.76;2.53	-0.58;2.30	0.292	-0.51	-1.75;0.72	-1.82;1.34	0.416
	CDrisksum_rp1	-0.24	-0.33;-0.16	-0.34; -0.16	<.001	-0.19	-0.24; -0.13	-0.28; -0.13	<.001
	Education (mid)[a]	0.02	-0.97;1.01	-1.08;0.99	0.972	-0.02	-0.72;0.67	-0.65;0.64	0.946
	Education (high)[a]	-0.17	-1.13;0.79	-1.13;0.76	0.733	0.67	-0.06;1.39	-0.06;1.53	0.073
	Age	-0.01	-0.10;0.08	-0.11;0.07	0.867	-0.03	-0.08;0.02	-0.07;0.01	0.239
	Smoking (no)[b]	-0.27	-1.56;1.02	-1.88;0.92	0.68	-0.39	-1.04;0.26	-1.08;0.26	0.237
	Moderation:								
	LTL x CD-RISC-10	-0.23	-0.59;0.13	-0.56;0.22	0.219	-0.04	-0.31;0.22	-0.53;0.31	0.749

Note: [a]The reference level is low. [b]The reference level is yes. The table only displays estimates for the interaction term in the moderation analysis. The moderation analysis included the same variables as the main effects model

Supplementary Table 8 Correlations among all study variables, including covariates.

Mothers											
	1	2	3	4	5	6	7	8	9	10	11
1 LTL	1										
2 EPDS	.09	1									
3 SCL	.08	.71***	1								
4 CD-RISC-10	-.07	-.46***	-.33***	1							
5 ACEs	-.03	.36***	.40***	-.15**	1						
6 Age	-.08*	-.06	-.03	.12*	.06	1					
7 Education	.01	-.04	-.05	.14**	-.08	.25***	1				
8 Smoking	.04	-.05	.00	.00	-.03	.07	.22***	1			
9 Income	.02	-.11*	-.09	.13*	-.03	.19***	.40***	.06	1		
10 Multipara	-.01	.07	-.07	-.03	.03	.17**	-.01	.02	-.20***	1	
11 Medication	.04	.03	.06	-.07	.05	-.06	.01	-.02	.04	-.04	1

Fathers										
	1	2	3	4	5	6	7	8	9	10
1 LTL	1									
2 EPDS	-.04	1								
3 SCL	-.08	.65***	1							
4 CD-RISC-10	.07	-.44***	-.30***	1						
5 ACEs	-.05	.31***	.30***	-.20***	1					
6 Age	-.10	.01	-.03	-.10	.08	1				
7 Education	.04	-.02	.01	.06	-.10	.14*	1			
8 Smoking	.13*	-.04	-.08	-.02	-.14*	.08	.19***	1		
9 Income	.03	-.18**	-.2***	.07	-.10	.24***	.25***	.13*	1	
10 Medication	.06	.12*	.18**	-.10*	.11	.04	-.05	-.07	-.03	1

*Note: The levels for education are 1: low, 2: mid, 3: high. The levels for smoking are 1: yes, 2: no. The levels for income are 1: <= 1500, 2: 1501-2500, 3: 2501-3500, 4: >3500. The levels for multipara are 1: nulliparous, 2: multiparous. The levels for medication are 0: no, 1: yes. ***p < 0.001, ** p <0.01, * p <0.05.*

References

1. Cawthon, R. M. (2009). Telomere length measurement by a novel monochrome multiplex quantitative PCR method. Nucleic Acids Research, 37(3), e21–e21. https://doi.org/10.1093/nar/gkn1027
2. Cawthon, R. M., Smith, K. R., O'Brien, E., Sivatchenko, A., & Kerber, R. A. (2003). Association between telomere length in blood and mortality in people aged 60 years or older. The Lancet, 361(9355), 393–395. https://doi.org/10.1016/S0140-6736(03)12384-7
3. Hellemans, J., Mortier, G., De Paepe, A., Speleman, F., & Vandesompele, J. (2007). qBase relative quantification framework and software for management and automated analysis of real-time quantitative PCR data. Genome Biology, 8(2), R19. https://doi.org/10.1186/gb-2007-8-2-r19
